# A Preclinical Pipeline for Translational Precision Medicine—Experiences from a Transdisciplinary Brain Tumor Stem Cell Project

**DOI:** 10.3390/jpm11090892

**Published:** 2021-09-07

**Authors:** Andres Vargas-Toscano, Christoph Janiak, Michael Sabel, Ulf Dietrich Kahlert

**Affiliations:** 1 Department of Neurosurgery, Medical Faculty, Heinrich-Heine University Düsseldorf, 40225 Düsseldorf, Germany; Michael.Sabel@med.uni-duesseldorf.de (M.S.); mail@ulf-kahlert.com (U.D.K.); 2 Institut für Anorganische Chemie und Strukturchemie, Heinrich-Heine-University Düsseldorf, 40204 Düsseldorf, Germany; janiak@uni-duesseldorf.de; 3 Molecular and Experimental Surgery, Department of General, Visceral, Vascular, and Transplant Surgery, University Hospital Magdeburg, 39120 Magdeburg, Germany

**Keywords:** precision medicine, translational research, drug screening, physical therapy of cancer, stem cells

## Abstract

Efficient transdisciplinary cooperation promotes the rapid discovery and clinical application of new technologies, especially in the competitive sector of oncology. In this review, written from a clinical-scientist point of view, we used glioblastoma—the most common and most aggressive primary brain tumor as a model disease with a largely unmet clinical need, despite decades of intensive research—to promote transdisciplinary medicine. Glioblastoma stem-like cells (GSCs), a special tumoral cell population analogue to healthy stem cells, are considered largely responsible for the progression of the disease and the mediation of therapy resistance. The presented work followed the concept of translational science, which generates the theoretical backbones of translational research projects, and aimed to close the preclinical gap between basic research and clinical application. Thus, this generated an integrated translational precision medicine pipeline model based on recent theoretical and experimental publications, which supports the accelerated discovery and development of new paths in the treatment of GSCs. The work may be of interest to the general field of precision medicine beyond the field of neuro-oncology such as in Cancer Neuroscience.

## 1. Introduction and Overview

The preclinical research gap generated between basic research mostly focused on scientific discoveries, and clinical work mostly focused on medical practice. This is one of the main reasons why several technological advancements are not translated into medical interventions for patients and communities [1,2]. In the current era of precision medicine, complex diseases like cancer and especially brain tumors require the rapid implementation of advanced technologies to develop effective treatments [3,4,5]. Thus, a theoretically ideal way to achieve this goal and bridge the gap is the integration of translational research and precision medicine. A good example of this integration in an oncological context is the merge of the Translational Research Working Group and the Personalised Medicine Task Force of the European Society for Medical Oncology (ESMO) into a single research group in 2014 [6].

Particularly, the complex field of glioblastoma has evidenced highly hierarchical behavior, and the most aggressive cells share equivalent characteristics and molecular markers with normal stem cells. Thus, these tumor cells with stem-like traits are referred to as glioblastoma stem-like cells (GSCs) and show a direct correlation between tumor aggressiveness and their degree of stemness or differentiation. This behavior is complemented by the highly invasive and proliferative pattern of cells that transition from a static epithelial-like behavior (proneural) to a higher invasive (mesenchymal) behavior through the process of epithelial-like-to-mesenchymal transition (EMT) [7,8]. Those cell biology and biochemistry properties of the cells are at least in part explaining their fatal malignant behavior [9,10,11,12,13].

The current work includes stem cell-derived disease models as a pathophysiologically relevant test platform. Subsequently, we apply integrative chemistry research on nano-pharmacology to develop improved drug delivery, robotic-assisted substance screening technology to develop individualized in vitro drug therapy resistance profiles, and the interrogation of physical therapy validation to support the rationale of new combination treatment regimens. In the context of validating our findings in the molecular datasets of patient samples, respecting ethnic and gender diversity, we believe that our work is of value to the general field of personalized medicine beyond the field of neuro oncology.

Ultimately, despite the importance of preclinical research, no explicit preclinical pipelines of integrated translational precision medicine focused on neuro-oncology or glioblastoma have been described in the literature to the best of our knowledge. Therefore, we designed this straight-forward pipeline based on the combination of recent literature with the published experimental results of a doctoral project from our laboratory in the context of GSCs, keeping in mind a translational, patient-oriented mindset to bridge the preclinical gap, accelerate the development of next-generation therapies for neurooncology and glioblastoma, and encourage clinicians and scientist to conduct translational preclinical research.

### 1.1. Translational Science and Research

Translational science transforms the empirical research process into a predictive, methodological branch of science [14]. This concept, carefully developed by the USA-National Center for Advancing Translational Sciences (NCATS) [14,15], establishes translational science as the logical metastructure, background process, or theoretical backbone of targeted translational research and translational medicine projects. These as a whole promise to close the gap of preclinical research and increase its reproducibility by coordinating every available scientific, clinical, industrial, and political-economical health resource to efficiently transform scientific discoveries into established medical interventions, which then are applied in the population through standardized pipelines or equivalent strategies to—on the one side—generate rapid and reproducible research results and—on the other—generate a transparent theoretical backbone for further translational research projects. This is summarized by the Translational Science Spectrum (TSS) model designed by the NCATS [1], sharing concepts with the European Society for Translational Medicine (EUSTM) [16]. These models are further integrated and aimed to be further developed during this review.

### 1.2. Precision Medicine in Cancer and Glioblastoma

Cancer is a highly adaptable, genetically complex disease. For this reason, traditional “one size fits all” approaches, such as broad-spectrum chemotherapies, have fallen short and are being more and more replaced by molecularly-tailored, highly targeted medical treatments and procedures [3,4,5]. This is the core of most precision medicine approaches. Originally, this concept was called ‘personalized medicine’, but due to misunderstandings, the ESMO and the National Research Council of the United States established the term “precision” as the new standard [3,17]. It is defined by the ESMO as a healthcare approach with the primary aim of identifying which interventions are likely to be of most benefit to which patients, based upon the features of the individual and their disease [3,17,18]. Applied to glioblastoma and GSCs, if we had an extensive knowledge of the molecular characteristics of the tumor in individual patients and know several available tools to target these specific features, we would theoretically ensure the development of highly efficient treatments with minimal adverse reactions. Two interesting examples of precision medicine in glioblastoma and cancer treatment are the GBM AGILE (Adaptive Global Innovative Learning Environment) [19] and the TESLA (Tumor Neoantigen Selection Alliance), a global precision medicine consortium for targeted tumor epitope immunotherapy [20].

## 2. Integrative Translational Precision Medicine Pipeline to Accelerate the Development of Next-Generation Therapies

### 2.1. Integrative Translational Precision Medicine Pipeline Overview

**Figure 1:**
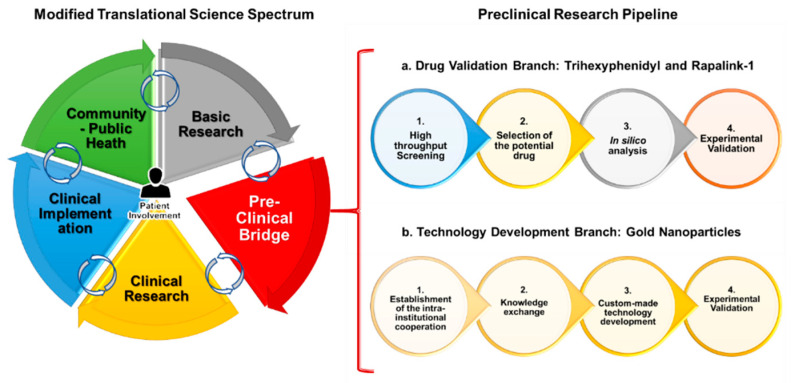
Modified translational science spectrum and integrated preclinical pipeline. On the left side of the figure, the different elements of the modified translational research spectrum are shown; on the right side, the branches of the integrated preclinical research pipeline are shown. (**a**) Drug validation branch and (**b**) technology development branch.

Preclinical biomedical research is a key step in the translational process, which acts as a connecting “bridge” between basic and clinical research. Namely, it requires the development of research projects that combine clinical experience with basic scientific knowledge, with the goal of providing solutions to a medical need. This was represented in Figure 1 by combining the concepts of the EUSTM and the NCATS-TSS to design a modified TSS (mTSS) that includes the following core steps: basic research, emphasized-preclinical research bridge, clinical research, clinical implementation (including commercial transfer), and community-public health. Those are non-linear, intertwined components that are designed to include the appreciation of perspective of the patients in every step of the way. Generally, the whole translational process would further lead to either the continuous progression of a research project or to a reversed or non-linear translational progression through all the steps of the mTSS.

Nevertheless, the focus of this review was specifically to describe our efforts to optimize the preclinical research bridge by enabling the generation of relevant experimental data. The pipeline was divided in a drug validation branch, which resulted in the identification of RapaLink-1 (RL1) and Trihexyphenidyl (THP) as anti-stem-cell effective chemotherapy candidates [21,22], and a technology development branch, where inorganic nanoparticles from a basic research laboratory were translated into preclinical research [23]. Both branches involved state of the art technologies, pathophysiologically relevant three-dimensional organoid-like 3D culture techniques, and either in vitro, ex vivo, or in silico models to serve as biological matrix for therapy and technology validation. Such organoids or organoid-like cells could then be further bio-banked for future extensive experiments, as previously described in the literature [24,25]. The mTSS and the preclinical pipeline, represented in Figure 1, consolidated the theoretical backbone of the current review.

### 2.2. Drug Validation Branch

a.1. High throughput screening

The first step of this pipeline branch was to perform a high throughput screening of a panel of 167 acknowledged blood-brain-barrier penetrating drugs already approved for human use. To this end, we programmed and parameter-optimized a local industry-grade robotic workstation in order to test the panel in an in vitro model of IDH-wt (Isocitrate dehydrogenase wildtype) GSCs. Importantly, in our experience, the pipetting-based system, which allows for the gentle and precise aspiration and dispersion of liquids, is more suitable to conduct screening assays with sensitive biological components such as suspension stem cell models as compared to printing-based screening systems. Considering cell growth inhibition as the main measured outcome, we identified 22 previously unrecognized repurpose-candidates to kill GSCs. Among them, THP, an anticholinergic drug used in the treatment against Parkinson’s disease, stood out as the most potent compound, for which we performed further manual mechanistic assays with this molecule. In addition, four other neurotransmitter-modulating agents were identified, providing an integrative link between neuroscience and neuro-oncology by demonstrating the utility of these molecules in the treatment of IDH-wt GSCs [21]. Given that the nerve-cell microenvironment is increasingly considered to play major roles in tumor progression and the therapy resistance of cancer outside the central nervous system, our results may be of interest to fields other than brain cancer [26]. Figure 1(a1).

a.2. Selection of the potential drug

Furthermore, given the fact that the first-generation Mammalian Target of Rapamycin (mTOR) inhibitor everolimus also stood out in our screening results, with a potent effect against GSCs, we chose to inhibit the mTOR pathway as the second step of this pipeline branch. This is mainly because it had a higher translational potential than the other molecules and because mTOR signaling is considered a hallmark of cancer, with various clinical trials on pharmacological mTOR inhibitors. This presents a more accepted intervention route [27,28,29]. We chose RL1, the most effective third-generation mTOR inhibitor, recently developed from a prestigious lab, to aim towards a complete pathway inhibition by targeting both mTORC 1 and 2 [30]. Figure 1(a2).

a.3. In silico analysis

Once the drug was selected, we performed an in silico analysis of clinical data from the international cancer genome atlas databases TCGA (The Cancer Genome Atlas) [31] and CGGA (Chinese Glioma Genome Atlas) [32]. Followed by the verification of these results in our in-house surgery-room-derived glioblastoma tissue samples [22]. Thus, we corroborated that mTOR inhibition was relevant in the context of clinical glioblastoma treatment and that this was correlated with EMT and stemness modulation in the respective tumor models. With this computational screen, we also ensured to include the diversity dimensions of our pipeline, namely ethnic, gender, and age diversity (mostly on patients over 18 years old) Figure 1(a3).

a.4. Experimental validation

Accordingly, we completed further experiments to ensure effective translation, including the validation of the effect of RL1 in GSCs in contrast to healthy human stem cell models. Here, we integrated neural stem cells derived from induced pluripotent cells as well as from fetal-derived neural stem cells. We believe that such off-target validation is particularly worth mentioning, both from the technology level as well as a possible disregarded confounder in many early drug development projects. Anti-stem cell signaling directed, conservative oncology clinical trials failed due to the intolerably high adverse effects of the test compounds on healthy stem cells of the patients [33]. Moreover, broad mode-of-action analyses were performed, finding a mainly anti-mitotic, partially pro-apoptotic effect of RL1 in GSCs. We further evidenced the synergistic effect of RL1 with the standard glioblastoma treatment temozolomide (TMZ) and the promising Tumor Treating Fields (TTFields) technology. Accordingly, the results of the identified interaction of RL1 with TTFields contributed to undergoing intellectual property developments with its manufacturing company, further advancing RL1 in the mTSS steps Figure 1(a4).

### 2.3. Technology Development Branch

b.1. Establishment of the intra-institutional cooperation

The first step of this pipeline branch, aimed at the development of GSC targeted local nanoparticles, was to establish an inter-institutional cooperation with the natural science faculty (inorganic biochemistry department). Based on our own experience, the interaction of medical clinics with basic natural science labs are relatively rare, particularly surgical-related departments. Despite the ongoing effort on adjusting the different “scientific languages” spoken in the two departments, we believe such interactions with very basic material science labs are of high innovative character. Figure 1(b1).

b.2. Knowledge exchange

The second step was to consolidate all of the required infrastructure through extensive knowledge exchange. Namely, defining the main desired combined objectives of our institutes, selecting the materials, establishing the safety/sterility experiments required for the use of the nanoparticles in a biological context, designing the experimental protocols, and implementing the first in vitro experiments. A specific learning experience is the incompatibility of the simplest in vitro assays commonly used in pure biological projects, such as the colorimetric- or bioluminescence-mediated quantification of cellular growth when working with the chosen inorganic compounds. To our surprise, many published studies seem to have overcome this problem somehow, but we had to adjust our assays to allow for the manual counting of cell colonies to score the therapy effect [23,34]. Figure 1(b2).

b.3. Custom-made technology development

The third step, once the infrastructure was established, was to actively customize this nanotechnology based on our extensive collaborative experiments and defined preclinical and basic research chemical objectives. For this, we defined the chemical synthesis procedure and materials and tested the cytotoxicity, uptake, and internalization of the nanoparticles in GSCs. Figure 1(b3).

b.4. Experimental validation

We eventually corroborated the effects of the most promising nanoparticle candidates in the GSC models by demonstrating biocompatibility and the efficient internalization levels (nanofection) on hard-to-transfect, living GSCs. Thus, on one side, we achieved the goal of functionally validating the potential and low toxicity of our locally manufactured nanocarrier-technology, and on the other side, we assembled a functional transdisciplinary working hub. This platform development already resulted in a promising follow-up project by the team, functionalizing the carrier platform with the promising anti-metabolic drug candidate CB 839 [34]. Figure 1(b4).

## 3. Discussion

Although traditional serendipity research models have been partially useful and necessary in the past, they require several years to generate results, extensive resources to reach the community, and they tend to be unpredictable in their course. In consequence, it is calculated that there are thousands of potentially effective treatments suitable for medical application, including drug candidates and novel technologies, that are stagnant within (or even before) the preclinical research gap [35]. Approximately, out of 5000 cancer compounds identified in basic research laboratories, only 250 enter preclinical testing. Out of these 250, fewer than 10 advance from the preclinical phase to clinical trials, and just about one will be approved by regulation authorities for the treatment of specific cancer types or diseases. The process of bringing a new treatment from the research stage to the clinic is estimated to take between 10–13 years [35]. Now, only about 500 human medical conditions have curative or established treatment among several thousand [1]. As most of the developments emerge in academic labs funded by public resources, the described scenario opposes a socio-economic and ethical burden on the taxpayer and patients.

This, therefore, supports the hypothesis that translation does not occur naturally or spontaneously and that it requires active, explicitly explained, and organized efforts to optimize the research process [1,14,15]. Thus, in order to generate novel efficient therapies to treat malignant cancers using modern precision medicine technologies, it is theoretically optimal to resort to the emerging field of translational science, which uses tools such as dynamic transdisciplinary maps, workflows involving clinicians at every stage of the translational process, and targeted pipelines to achieve this purpose. Ideally, encouraging other doctors and scientists of any field to develop pipelines or similar translational models to reduce the preclinical research gap and increase the scientific interconnectivity of future research projects.

Moreover, translational research in our hands requires the interrogation of a suitable quality control system regulating the documentation, functionality, authenticity, and decontamination of the lab tools and the achieved results [36]. Digitalization in a lab environment further supports the transparency and translational potential [37].

Examples of previous preclinical research pipelines in the context of medicine and cancer would be *The global preclinical antibacterial pipeline* by Theuretzbacher et al. [38] and *The global pipeline of cell therapies for cancer* by Yu et al. [39]. Nevertheless, to the best of our knowledge, there is only limited content available that intrinsically describe the integration of out-of-scratch development procedures in clinical-surgical centers and only requires minimal resources. Although not related to stem cells or glioblastoma, the publication by McCarthy et al. 2020 [40] regarding immuno-oncology model systems is a useful source for designing pipelines for precision medicine. The current review included an integrative translational science pipeline, which may then be applied either to other scientific contexts or integrated into broader encompassing dynamic maps within collaborative translational science efforts to progress through the spectrum [14,15]. An example of such a very powerful and successful dynamic map is the ‘Drug Discovery, Development and Deployment Map’ by Wagner et al., which integrates the complexity of small-molecule drug development [41].

An optimal example of a coordinated translational precision medicine effort is the COVID-19 (Coronavirus Disease 2019′) vaccine development pipeline during 2020 and 2021. Through this process, a novel technology previously used for cancer was swiftly repurposed, adapted, and tested in order to have an approved product in less than a year [42]. This set a historical precedent in medical product development. The authors acknowledge the massive amount of funding in a global coordinated initiative as the basis of such efficient developments and that this may only be suitable for world population emergencies, such as a pandemic with a life-threating virus.

Our drug discovery branch identified a high potency compound designed for the neurotransmitter-modulating mode of action. Since we worked in model systems that only comprise tumor cell population, we believe our results may be based on an off-target effect of the drug. Evaluation is currently ongoing. Nevertheless, we think our pragmatic attempt may also classify and possibly contribute to a completely new field of cancer research, especially in connection to central nervous system tumors. Glioblastoma cells are considered to require the parallel neurotransmitter activity of their neighboring neurons. This phenomenon is reported as *neuro-glioma synapses* [43,44]. It basically describes how neurons and brain cancer cells form excitatory synapses and generate an electrically active tissue that signals other glioma cells in the network to promote their migration and growth. With the effect of THP on the cancer neuroscience micro and macro environment, it possibly even influencing the tumor immune microenvironment seems plausible. In this regard, these results may be a starting point to bridging the preclinical gap through the integration of in vitro pharmacology screening with basic neuroscience and allowing for the future use of technologies such as multiplexed spatial and temporal tissue imaging to understand this active phenomenon [45].

Although transcriptional subtypes of glioblastoma are accepted as molecular sub-identities of this disease [7], recent high-profile work identified that the stem cell population of this tumor consists of four plastic cellular and molecular defined stages [46]. We acknowledge that from a translational point of view, and to validate our results on the current stage of science, it would be highly desirable to classify our models for the composition according to Neftel et al. [46]. However, we cannot offer that piece of data at the moment. Nevertheless, as our models were validated by various clinically relevant markers of neuro pathological diagnostics such IDH1 wildtype [22,46], and given the fact of their proven in vivo tumorigenicity, we hypothesize the high clinical relevance of our model systems. We acknowledge that although working with 3D human model systems, in vitro research cannot fully appreciate the complexity of disease. The validation of experiments in animal models, such as xenograft tumor models in immune compromised mice or immune tolerant mice of human tumors [47], are required to complete a thorough preclinical pipeline. Interestingly, model systems of different stages of the clinical development of cancers are missing. This is a big problem for the field, representing the unavailability of pathophysiologically relevant, patient-matched comparator models that resemble early versus late stages of the pathology. Synthetic cancer cell models, based on the stepwise introduction of genetic elements resembling tumor malformation in a healthy stem cell matrix, are emerging as a technology combating this problem [48]. Moreover, surgical resection from the primary tumor and recurrent tissue from one patient to established patient-derived models from different time points of the disease may be feasible but needs the dedication of surgeons and lab personnel to join forces. In our experience, this is only possible in specialized academic treatment centers with coherent patient follow up procedures in place.

We performed a targeted data mining in silico assessment, employing the data from the mentioned TCGA and the more recent CGGA [32], in which we validated the relevance of the mTOR pathway in a glioblastoma stemness and EMT context. With this attempt, we secured a direct translational jump by using community patient data from different independent sources and enabling these results to possibly be correlated with novel technologies such as intravital imaging to monitor the tumor cell stemness or migratory behavior [45]. Guided by a clinical perspective, we chose the potent mTOR inhibitor RL1 as drug candidate with high anti-brain tumor efficacy but missing knowledge on its effectivity on GSCs. Strikingly, our results revealed its therapeutic anti-stem cell, anti-EMT effects against multiple GSC models. These effects are probably attributed to the strong inhibition at low nM concentrations of the full mTOR pathway through the mTORC-1 and mTORC-2 proteins. This closes the gap left by first generation inhibitors and promises a high predictive value of clinical efficiency with lower chances of resistance [49].

Moreover, as we identified the synergistic effect of RL1 when combined with TTFields and TMZ, we even evidenced the benefit of this treatment strategy in a model representing one of the most challenging manifestations of the disease, namely an IDH-wt, unmethylated MGMT (O6-methylguanine-DNA methyltransferase), GSC cell line. Ultimately, by showing fewer toxic effects on non-cancer stem cell controls, and given the results and previous report on the effectiveness of RL1 in animal models of human glioblastoma [30], RL1 was thus established as a promising candidate for clinical trials and progression through the glioblastoma mTSS.

On the technology development branch, we focused on nanoparticles, which are actually very common occurrences in nature and in our daily lives [50,51,52]. However, only with the advancement of technology have scientists been able to build and customize highly efficient nanoparticles for medical purposes [53]. Probably, the most recent and notable example is the effective RNA-lipid-nanoparticle vaccines developed against the COVID-19 virus in 2020 [54]. In the context of cancer research, their main uses range from targeted drug delivery, gene therapy, hyperthermia, radiation therapy, diagnostic mechanisms, and controlled release, among others [55]. Furthermore, there are currently numerous ongoing nanoparticle-based therapies in clinical trials [56].

We specifically focused on AuNP because they are robust and biocompatible vessels for research, treatment, and diagnosis in the medical field [57,58]. Some of the main, currently used examples of AuNP in oncological clinical trials are the NU-0129 RNA interference drug, delivered on spherical AuNP for the treatment of glioblastoma, and the AuroLase drug, consisting of polyethylene-glycol-coated silica-gold nanoshells for near infrared light thermal ablation in prostate cancer.

Therefore, working closely with the natural science workforce, we reproducibly synthesized fluorescent AuNP with optimal-sized cores between 3 and 6 nm, further developing a novel synthesis strategy called the “one-pot method” and achieving fluorescent AuNP in a rapid, inexpensive, and simple manner. Furthermore, we established these nanoparticles as practical, biocompatible, and nanofection therapy vessels against GSCs. Finally accomplishing the pipeline branch goal of translating a basic science nanotechnology into a promising preclinical research nano-carrier for pharmacotherapy, we call for the further assessment of its utility in gene therapy or equivalent future applications in the context of GSCs. We developed a novel, locally made product and a strong research collaboration backed by multiple specifically assigned researchers who continue to work on this project [23,34]. However, the data evaluation of very specialized experiments investigating dynamic interacting networks on the single cell level such as single cell omics or immune repertoire sequencing—as well as nucleic acid sheddome of tumors to influence the cancer environment, thereby contributing to emergence of therapy resistance—must benchmark the durable effects of future nanotherapies.

## 4. Conclusions

Translational-oriented biomedical research project design is particularly suitable for academic clinical-surgery labs, offering a high chance for productive and promising preclinical support for optimizing the treatment of the respective patient cohorts.

We theoretically and experimentally validated the integrative translational precision medicine pipeline as a strategy to bridge the preclinical research gap, generating targeted GSC therapies with high translational potential to become next-generation treatments against glioblastoma and brain tumors. This is the first one reported in the literature, to the best of our knowledge. Consequently, we encourage basic and clinical scientists to integrate precision medicine with translational research by explicitly publishing their translational logic through a pipeline or an equivalent model. Hypothetically, this can foster the accelerated development of novel precision medicine-based treatments in the glioblastoma and neuro-oncology fields, extending the replicability of their experimental backbone design and results to different scientific branches towards their integration into future complex dynamic maps within a transdisciplinary, collaborative, and translational science model.
